# Supplementary material for: Time‑resolved multi-omic analysis of paclitaxel exposure in human iPSC‑derived sensory neurons unveils mechanisms of chemotherapy‑induced peripheral neuropathy
Source: Cell Death Dis. 2026 Feb 10;17(1):211. doi: 10.1038/s41419-026-08445-2 (PMC12921266; doi:10.1038/s41419-026-08445-2)
Supplement: Supplementary file 15 — Dataset 1. [file 41419_2026_8445_MOESM15_ESM.zip › CDD_Open_Data/04_RNA_Seq/Timeline_panel_plots_etc_2023-01-23_1609.zip!/Panel_Plots_etc/Evidence_Plots/6h/evidence_plot_standard contrasts_msigdb_hallmark_6h_PTX_vs_DMSO_ID2_M5936_HALLMARK_OXIDATIVE_PHOSPHORYLATION.pdf]

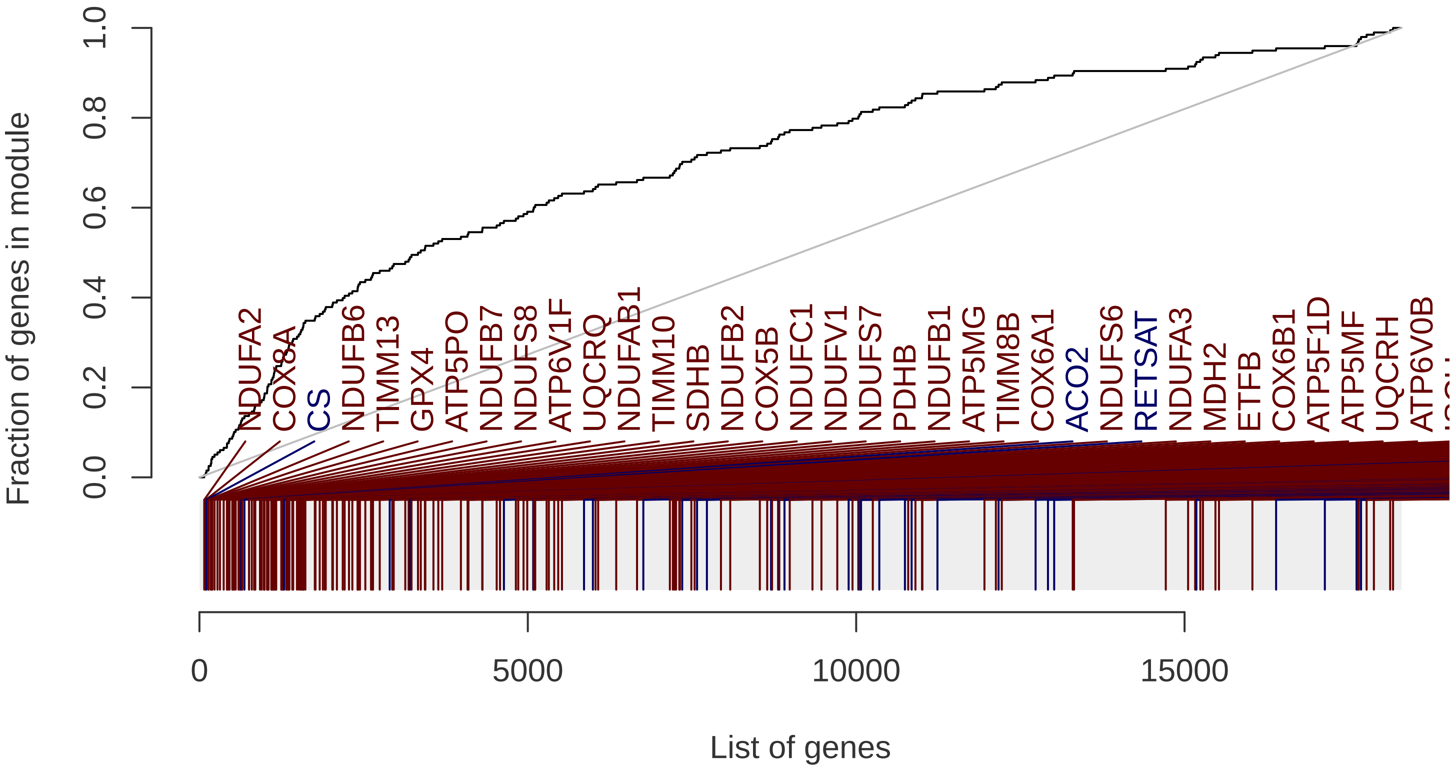

Fraction of genes in module
0.0
0.2
0.4
0.6
0.8
1.0
NDUFA2
COX8A
CS
NDUFB6
TIMM13
GPX4
ATP5PO
NDUFB7
NDUFS8
ATP6V1F
UQCRQ
NDUFAB1
TIMM10
SDHB
NDUFB2
COX5B
NDUFC1
NDUFV1
NDUFS7
PDHB
NDUFB1
ATP5MG
TIMM8B
COX6A1
ACO2
NDUFS6
RETSAT
NDUFA3
MDH2
ETFB
COX6B1
ATP5F1D
ATP5MF
UQCRH
ATP6V0B
0
5000
10000
15000
List of genes
